# Co-occurrence of multiple cerebral infarctions due to hypercoagulability associated with malignancy and meningeal carcinomatosis as the initial manifestation of gastric cancer

**DOI:** 10.1186/s12883-014-0160-9

**Published:** 2014-08-08

**Authors:** Akiko Kawasaki, Keisuke Suzuki, Hidehiro Takekawa, Toshiki Nakamura, Masanari Yamamoto, Yohei Asakawa, Madoka Okamura, Koichi Hirata

**Affiliations:** 1 Department of Neurology, Dokkyo Medical University, 880 Kitakobayashi, Mibu, Shimotsuga 321-0293, Tochigi, Japan; 2 Department of Neurology, Rehabilitation Amakusa Hospital, Koshigaya, Saitama, Japan

**Keywords:** Multiple cerebral infarctions, Disturbance of consciousness, Meningeal irritation, Trousseau’s syndrome, Meningeal carcinomatosis, Gastric cancer

## Abstract

**Background:**

Meningeal carcinomatosis and hypercoagulability associated with malignancy are typical late stage complications in cancer patients. The co-occurrence of meningeal carcinomatosis and cerebral infarction related to hypercoagulability associated with malignancy in an individual as the initial manifestation of malignancy has not been previously reported.

**Case presentation:**

Herein, we report the case of an 80-year-old patient who presented with meningeal carcinomatosis and hypercoagulability related to malignancy as the initial manifestation of occult gastric cancer. The patient displayed consciousness disturbance, mild left facial paralysis, and bilateral positive Babinski’s sign. Using brain magnetic resonance imaging, the patient was diagnosed as having acute multiple cerebral infarctions. Cerebrospinal fluid (CSF) cytology showed adenocarcinoma and upper gastrointestinal endoscopy disclosed scirrhous gastric cancer. The patient presented with headache, fever, and meningeal irritation with a subacute course. Tuberculous or fungal meningitis was initially suspected; however, cytological evidence of adenocarcinoma in the CSF led to the diagnosis of meningeal carcinomatosis.

**Conclusion:**

The comorbidity of hypercoagulability associated with malignancy and meningeal carcinomatosis should be considered in a patient presenting with multiple cerebral infarctions, progressive disturbance of consciousness, fever, and meningeal irritation.

## Background

Meningeal carcinomatosis is characterized by diffuse invasion of tumor cells in the leptomeninges and cerebrospinal fluid [1], which occurs in 3-8% of all cancer patients. Among patients with any type of solid tumors, the highest incidence of meningeal carcinomatosis has been reported in patients with breast cancer (12-34%) and lung cancer (10-26%) [2], while the occurrence of meningeal carcinomatosis is rare in patients with gastric cancer with a reported incidence of 0.06-0.17% [3],[4]. Nevertheless, in autopsy studies, evidence of meningeal involvement was found in approximately 20% of cancer patients with neurological signs and symptoms [1],[5].

Malignant tumors, such as lung cancer, prostatic cancer, brain tumors, gastrointestinal cancer, uterine cancer, and ovarian cancer [6], are often associated with excessive coagulation; arterial and venous thrombosis is also a frequent complication [7]. Meningeal carcinomatosis and hypercoagulability associated with malignancy are treatment-resistant complications of malignancy. The early detection of both conditions is clinically important. However, the co-occurrence of meningeal carcinomatosis and cerebral infarction due to hypercoagulability associated with malignancy in the same individual, especially as the early manifestation of malignancy, has not been previously reported. Herein, we report a patient who presented with meningeal carcinomatosis and cerebral infarction due to hypercoagulability related to malignancy, which were the initial manifestations of occult gastric cancer.

## Case presentation

An 80-year-old woman developed unsteadiness of gait and required assistance to walk one week after she noted lower back pain. One week later, the patient presented with headache, nausea, and low-grade fever and was admitted to a local hospital. Acute multiple cerebral infarctions were diagnosed via brain magnetic resonance imaging (MRI), and anti-coagulant therapy was initiated. However, despite continuing medical treatment for 7 days, consciousness disturbance developed and she was transferred to our hospital. At the time of admission, the patient was 155 cm tall and weighed 61 kg. Her body temperature was 37.4°C, her blood pressure was 145/85 mmHg, and her pulse rate was 66/min. There was no blood pressure differential between the right and left arms. The physical examination, including palpations of the lymph nodes, chest and abdomen, was unremarkable. There was mild edema in the lower legs. Neurological examination revealed mild consciousness disturbance with a Glasgow Coma Scale (GCS) of 12 (E3V4M5), mild left facial paralysis, and bilateral positive Babinski’s sign. The patient displayed no motor weakness or sensory disturbances. Marked nuchal stiffness and a positive Kernig’s sign were observed.

**Figure 1:**
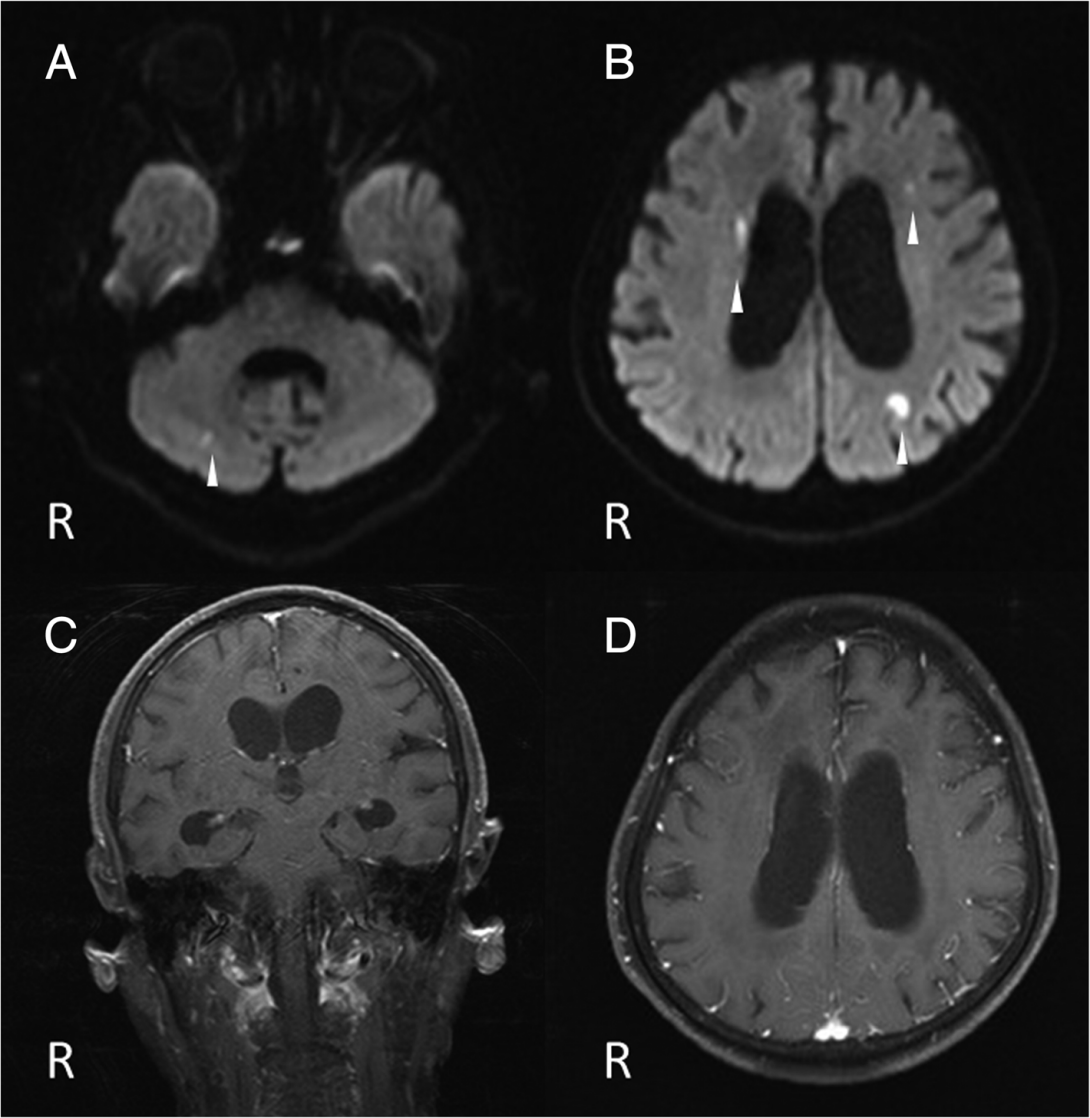
**Brain magnetic resonance images. A**, **B**; Diffusion-weighted images show high signal intensities in the right cerebellar hemisphere and in the deep white matter adjacent to the bilateral lateral ventricles and subcortices (arrowheads). **C**, **D**; Post-contrast T1-weighted images reveal no meningeal enhancement.

The patient’s laboratory data showed a white blood cell count of 12,100/μL with 87.3% neutrophils, C-reactive protein levels of 11.69 mg/dL, and D-dimer levels of 18.1 μg/mL. Cerebrospinal fluid (CSF) analysis disclosed 5 mononuclear cells/μL, a glucose level of 42 mg/dL (CSF-blood glucose ratio: 0.35), and a protein value of 46 mg/dL (Table 1). An electroencephalogram revealed frequent bilaterally asynchronous sharp wave activities. Brain MRI revealed hydrocephalus, and high signal intensities in the right cerebellar hemisphere, corona radiata, caudate nucleus, and the left parietal lobe on diffusion-weighted imaging (Figure 1A, B), with corresponding reduced apparent diffusion coefficient maps. No enhancement was observed in these lesions or the meninges on post-contrast T1-weighted images (Figure 1C, D). MR angiography showed intracranial vessel irregularities and stenoses of the right middle cerebral artery and vertebral artery. Electrocardiography displayed a sinus rhythm. Neither a potential cardiac source of embolism nor a source of the right-to-left shunt, including patent foramen ovale, was demonstrated via transthoracic and transesophageal echocardiography. A computed tomography scan of the chest detected calcification of the aortic arch. The patient was treated with intravenous heparin.

**Table 1:** CSF examination results

Cells	5	/μL
Mononuclear	5	/μL
Polymorphonuclear	0	/μL
Glucose	42	mg/dL
Protein	46	mg/dL
LDH	20	U/L
Lactate	32	mg/dL
Adenosine deaminase	<2.0	U/L
CSF-blood glucose ratio	0.35	

**Figure 2:**
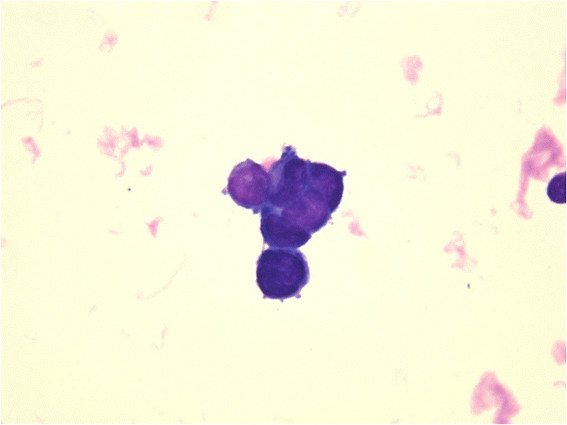
**The results of CSF cytology.** The cytology obtained from the CSF reveals adenocarcinoma.

The subacute clinical course, marked nuchal stiffness, and mild mononuclear pleocytosis with a decreased CSF-blood glucose ratio (Table 1) led us to consider meningoencephalitis due to tuberculosis or fungi; subsequently, antitubercular and antifungal drugs were administered. However, the CSF exams resulted in a negative cryptococcus antigen test and a low adenosine deaminase level (<2.0 IU/L). Additionally, CSF culture for bacteria, acid-fast bacilli and fungi was negative. The CSF cytology on admission revealed adenocarcinoma (Figure 2). Tumor markers were within normal ranges. The chest, abdominal, and pelvic computed tomography and gallium scintigraphy findings showed no evidence of malignancy. However, upper gastrointestinal endoscopy disclosed scirrhous gastric cancer, which was pathologically diagnosed as adenocarcinoma (signet-ring cell). The final diagnosis was meningeal carcinomatosis and excessive coagulation related to occult gastric cancer. Following discussion with the patient’s family, and based on the patient’s age, worsening consciousness levels (GCS of 6 on week 3), and poor performance status, she was transferred to the other hospital 22 days after admission, and supportive care rather than active management was administered.

## Discussion

In this report, we present the first case of the co-occurrence of cerebral infarction due to hypercoagulability associated with malignancy and meningeal carcinomatosis as the initial manifestation of occult gastric cancer. Our patient displayed headache, fever, and meningeal irritation with a subacute course. Although tuberculous or fungal meningitis was initially suspected, cytological evidence of adenocarcinoma in the CSF led to a diagnosis of meningeal carcinomatosis. Nonbacterial thrombotic endocarditis (marantic endocarditis) and infective endocarditis [8] or bacterial meningoencephalitis [9] can present with fever and acute multiple cerebral infarctions. However, in the patient reported here, no vegetation was detected via transthoracic or transesophageal echocardiography, and the CSF bacterial culture was negative. Hypercoagulability was likely the cause of cerebral infarction in our patient.

Meningeal carcinomatosis usually occurs in a patient who has a malignancy of known origin during the disease course and is typically a relatively late complication. The diagnosis can be made on the basis of CSF cytological findings. The sensitivity of the diagnosis for meningeal carcinomatosis increases as CSF cytology tests are repeated and the results are confirmed. The prognosis of untreated meningeal carcinomatosis is reported to be poor, with a median survival of 4–6 weeks from diagnosis [1]. Additionally, the type of primary cancer is a major prognostic factor. In a series of 90 patients with meningeal carcinomatosis who received focal irradiation and intraventricular methotrexate, 61% of the patients with breast cancer showed neurological improvement with a median survival of 7.2 months, while 39% of the lung cancer patients showed neurological improvement with a median survival of 3–4 months [10]. However, although rare, meningeal carcinomatosis can be the initial and early manifestations of the malignancy (as was observed in our patient) [3],[11]–[18]. In these patients, the diagnosis of meningeal carcinomatosis contributed to the identification of the primary malignancy and the primary tumors were found in the stomach [3],[12], the lung [14],[16], the bronchus [15] and the colon [13]. Common neurological presentations are headache [3],[11],[15] and bilateral deafness [12]–[14]. Radiation therapy and chemotherapy including intrathecal methotrexate are used in patients with meningeal carcinomatosis [3],[11],[12],[15],[16]. However, the prognosis remains poor, with a survival of 0.4-12 months from diagnosis. Cerebral infarction, which was likely due to tumor embolism and complicated with meningeal carcinomatosis, has been reported [11]. In contrast, our patient displayed cerebral infarction due to hypercoagulability associated with malignancy. High-grade fever may be uncommon.

Our patient displayed multiple cerebral infarctions caused by excessive blood coagulation, which was confirmed by elevated D-dimer levels and was related to the presence of a malignant tumor. Hypercoagulability syndrome associated with cancer or malignancy-related thromboembolism is also known as Trousseau’s syndrome [19].

Regarding the mechanism of the hypercoagulability state in cancer patients, tumor necrosis factor, interleukin-1 and interleukin-6, which are released by monocytes or macrophages, lead to endothelial damage. Additionally, interactions between tumor cells and macrophages activate platelets, factor XII, and factor X, leading to the generation of thrombin and thrombosis [7]. Based on the imaging findings in our patient, we concluded that the cerebral infarction was due to hypercoagulability and not due to the meningeal invasion of malignant cells. Therefore, meningeal carcinomatosis may have had a significant impact on the clinical course of this patient; however the role of meningeal carcinomatosis in the development of cerebral infarction was unclear in our patient. These co-occurrences may be coincidental.

Embolism is thought to be a common cause of stroke in cancer patients. Cestari et al. reported that embolic stroke is responsible for 54% of the strokes observed in cancer patients, whereas atherosclerosis accounts for 22% of these strokes [20]. Nonbacterial thrombotic endocarditis is characterized by the deposition of thrombi on previously undamaged heart valves in the absence of a bloodstream bacterial infection, which likely results from the hypercoagulable state and increased levels of cytokines associated with cancer [21]. In an autopsy study, nonbacterial thrombotic endocarditis (18.5%) and intravascular coagulation (9.6%) were the most common etiologies for cerebrovascular disease in cancer patients [22].

The incidence of stroke in patients with cancer is reported to be 0.12% with lung cancer as the most common primary lesion, followed by brain tumor and prostatic cancer. Metastasis to the brain or meninges was found in 6% of the cancer patients who developed stroke; among these patients, 2% had metastases to the central nervous system, as detected following the stroke onset. In a hypercoagulability state related to malignancy, removal of the causative tumor is the primary approach; however, this is difficult in many patients, including our patient, particularly during late-stage malignancy. A hypercoagulability state involves multiple mechanisms, and heparin use is recommended because it has the ability to irreversibly inactivate both activated factor Xa and thrombin, interrupt fluid-phase thrombosis and suppress secondary platelet activation [19]. Therefore, we administered heparin to our patient; however, the recurrence of cerebral infarction could not be prevented. The prognosis of a hypercoagulability state related to malignancy is poor. A 1-year survival rate of 12% was observed in patients who received a diagnosis of cancer simultaneously with or following an episode of venous thromboembolism [23]. A large study including 1,874 cancer patients found that the median survival following venous thrombosis and arterial thrombosis was 16.7 months and 7.7 months, respectively [24]. Additionally, in a recent study including 263 cancer patients with ischemic stroke, the rates of recurrent thromboembolism were 21%, 31%, and 37% at 1, 3, and 6 months, respectively; adenocarcinoma histology was independently associated with recurrent thromboembolism [25].

## Conclusion

We present the first case report of the co-occurrence of hypercoagulability related to malignancy and meningeal carcinomatosis as the initial manifestation of occult gastric cancer. Although rare, the comorbidity of hypercoagulability related to malignancy and meningeal carcinomatosis should be considered in a patient showing multiple cerebral infarctions, progressive disturbance of consciousness, fever, and meningeal irritation.

### Consent

Written informed consent was obtained from the patient’s son for the publication of this case report and any accompanying images.

## Competing interests

The authors declare that they have no competing interests.

## Authors’ contributions

AK and KS contributed to the diagnosis and treatment of the patient, and drafted the manuscript. HT contributed to the diagnosis and treatment of the patient and revised the manuscript. TN contributed to the diagnosis of the patient and critically revised the manuscript. MY, YA, and MO contributed to the diagnosis and treatment of the patient. KH contributed to revision of the manuscript and supervised this study. All of the authors have read and approved the final manuscript.
